# Effects of Motor-Evoked Potential Monitoring During Spine Surgery on the Length of Hospital Stay and Postoperative Acute Pain

**DOI:** 10.7759/cureus.92818

**Published:** 2025-09-21

**Authors:** Fukuyo Taichi, Yuta Mitobe, Yasuko Baba, Naoko Tachibana, Tetsuya Miyashita

**Affiliations:** 1 Perianesthesia Nursing, International University of Health and Welfare Graduate School, Tokyo, JPN; 2 Anaesthesiology, International University of Health and Welfare Mita Hospital, Tokyo, JPN; 3 Anaesthesiology, International University of Health and Welfare Narita Hospital, Chiba, JPN

**Keywords:** acute postoperative pain, general anesthesia, length of hospital stay, mep, spine surgery, tiva

## Abstract

Background: In spine surgery, intraoperative motor-evoked potential (MEP) monitoring is a crucial safety measure, helping to prevent and detect nerve injuries early during surgical operations. When performing intraoperative MEP monitoring, anesthesia management with total intravenous anesthesia (TIVA) is recommended. This study aimed to determine whether the introduction of MEP monitoring and TIVA contributes to a reduction in the length of hospital stay and postoperative acute pain in patients undergoing spinal surgery under general anesthesia.

Methods: One hundred and fifty-six patients, who underwent spinal surgery under general anesthesia in the operating room of Mita Hospital of International University of Health and Welfare between January 1, 2017, and March 30, 2023, were included. The patients were classified into two groups, the non-MEP monitoring group (OUT group) and the MEP monitoring group (INT group), and compared based on five categorical endpoints. This study is a retrospective observational study using electronic medical records. Note that no prior sample size calculation was performed in this study.

Results: One hundred patients were included in the analysis. Of these, 50 patients were classified into the OUT and INT groups for analysis. The INT group showed a significantly lower intraoperative fentanyl dosage (*p*=0.0189), a significantly lower proportion of intraoperative non-narcotic analgesic measures (*p*<0.001), and a significantly shorter operative time (*p*=0.0432) and significantly had fewer patients complaining of postoperative pain within 30 min (*p*=0.0186) and requiring analgesic measures within 24 h after surgery (*p*=0.0334); length of hospital stay was also significantly reduced (*p*=0.0282).

Conclusions: The introduction of MEP monitoring in spinal surgery under general anesthesia was associated with reduced acute postoperative pain and shorter hospital stay, potentially through improved surgical efficiency and appropriate intraoperative opioid management.

## Introduction

In spine surgery, intraoperative motor-evoked potential (MEP) monitoring is a crucial safety measure, helping to prevent and detect nerve injuries early during surgical operations [1-6]. When performing intraoperative MEP monitoring, anesthesia management with total intravenous anesthesia (TIVA) is recommended [7]. MEP monitoring was introduced in April 2017 for all spinal surgeries. Therefore, we switched from conventional general anesthesia with volatile inhalation anesthetics to TIVA [8-11]. This study aimed to determine whether the introduction of MEP monitoring and TIVA contributes to a reduction in the length of hospital stay and postoperative acute pain in patients undergoing spinal surgery under general anesthesia.

## Materials and methods

This retrospective observational study utilized electronic medical records and was conducted with the approval of the Ethics Review Committee of the International University of Health and Welfare, Mita Hospital and Tokyo Akasaka Campus (Approval No.: 23-Ig-163, January 24, 2024). In compliance with the “Declaration of Helsinki” and the “Ethical Guidelines for Life Sciences and Medical Research Involving Human Subjects,” this study was conducted using an opt-out method, ensuring research participants were informed in advance and had the opportunity to decline participation. The full text of the disclosure document was posted on the website of the Department of Anesthesiology, International University of Health and Welfare, Mita Hospital, allowing participants to review relevant study information. Note that no prior sample size calculation was performed in this study.

Study population

**Figure 1:**
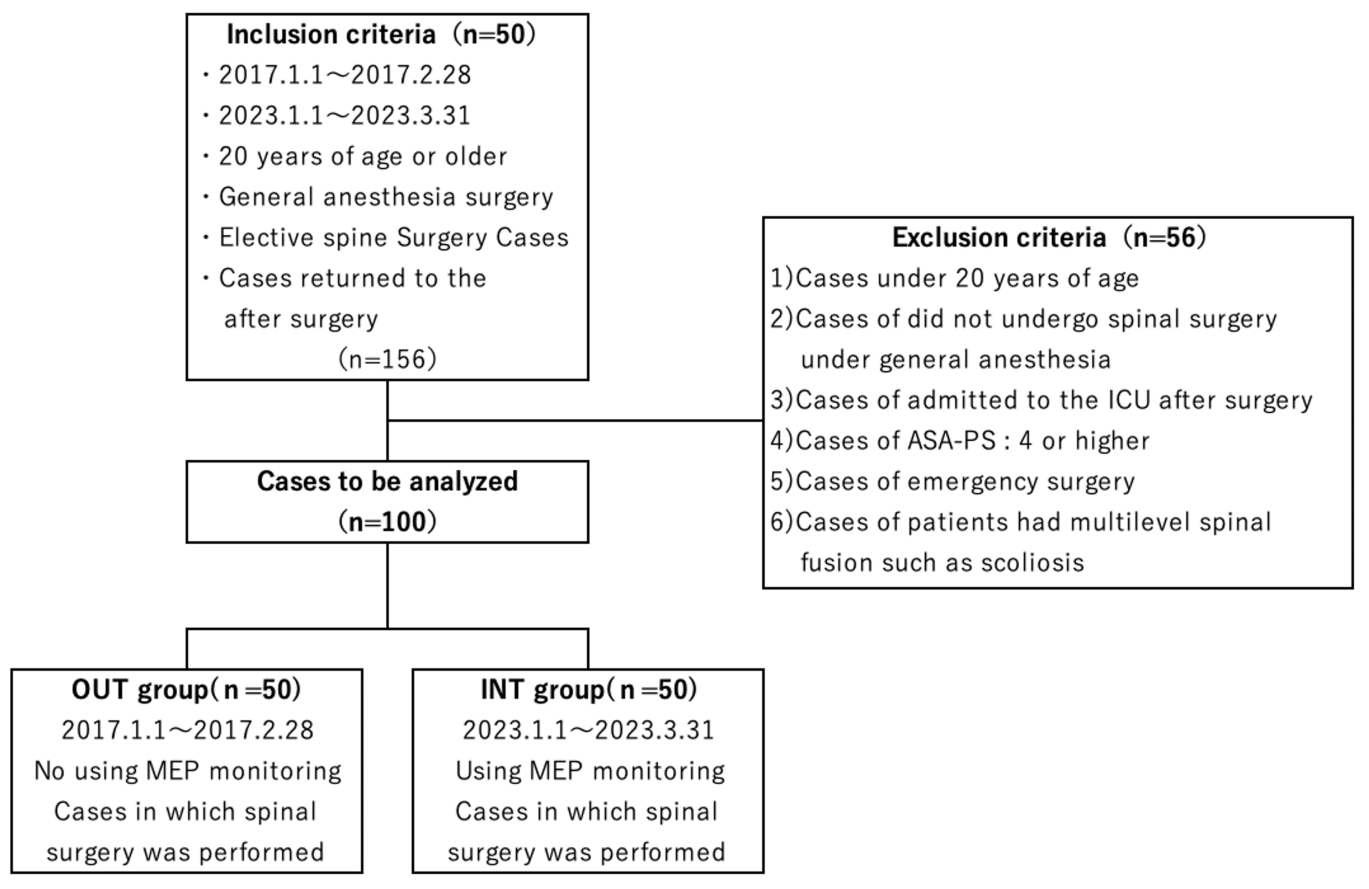
Flowchart of analysis study population. MEP: Motor-evoked potential

The study population is shown in Figure 1. Patients, aged 20 years and older, who underwent spinal surgery under general anesthesia in the operating room of Mita Hospital of International University of Health and Welfare between January 1, 2017 and March 30, 2023 were included. Patients who underwent elective spinal surgery under general anesthesia without MEP monitoring between January 1, 2017 and February 28, 2017 and returned to the general ward after surgery were defined as the non-MEP monitoring group (OUT group). Patients who underwent elective spinal surgery under general anesthesia with MEP monitoring between January 1, 2023 and March 30, 2023 and returned to the general ward after surgery were defined as the MEP monitoring group (INT group). Patients were excluded from the analysis based on the following six criteria: (i) under 20 years of age, (ii) did not undergo spinal surgery under general anesthesia, (iii) admitted to the intensive care unit (ICU) after surgery, (iv) had an American Society of Anesthesiologists Physical Status (ASA-PS) score of 4 or higher, (v) underwent unscheduled emergency surgery, and (vi) had multilevel spinal fusion such as scoliosis.

Evaluation of preoperative factors

The following preoperative factors were evaluated: patient’s age at time of surgery, sex, height, weight, body mass index (BMI), history of alcohol consumption, smoking, allergies, surgery, and medications, medical history, primary illness, surgical site (cervical, thoracic, or lumbar spine), risk of anesthesia-related complications, and abnormal physiological tests immediately before surgery. Blood tests included total protein (TP), albumin (Alb), hemoglobin (Hb), hematocrit (Hct), creatinine (Cre), estimated glomerular filtration rate (eGFR), aspartate aminotransferase (AST), alanine aminotransferase (ALT), white blood cell (WBC), platelets (PLT), prothrombin time (PT), activated partial thromboplastin time (APTT), fibrinogen degradation products (FDPs), and C-reactive protein (CRP). Blood tests were performed immediately before surgery.

Evaluation of intraoperative factors

The following intraoperative factors were evaluated: ASA-PS, anesthesia method, operation time, in/out balance, dose of intraoperative analgesia, various anesthesia-related drugs, and dose of intravenous patient-controlled analgesia (IV-PCA).

Evaluation of immediate postoperative factors (within 30 min after surgery)

The following immediate postoperative factors were evaluated within 30 min after surgery: pain levels, pain management methods, adverse events, and medical interventions. The types of drugs used were classified into five categories: no drug use, acetaminophen single drug use, non-steroidal anti-inflammatory drug (NSAID) single drug use, morphine single-drug use, and multi-drug combination use.

Evaluation of postoperative factors

The following postoperative factors were evaluated: length of hospital stay, vital signs (temperature, pulse rate, mean blood pressure, respiratory rate, and transcutaneous oxygen saturation), Numerical Rating Scale (NRS), and types and doses of drugs used up to 24 h after surgery. The types of drugs used were classified into five categories: no drug use, acetaminophen single-drug use, NSAID single-drug use, morphine single-drug use, and multi-drug combination use. The NRS was also evaluated on a 6-point scale from 0 to 6, instead of 11, on a scale of 0 to 10.

Analgesics used up to 24 h after surgery

The following analgesics used up to 24 h after surgery were evaluated: total use (mg) of acetaminophen, NSAIDs, tramadol, and pentazocine.

Statistical analysis

First, continuous variables in the evaluation items were tested for normal distribution using the Shapiro-Wilk test. Normally distributed continuous variables are specified as means (standard deviations), and non-normally distributed continuous variables are specified as medians. Univariate analyses were conducted using Fisher's exact test for normally distributed continuous variables and the Mann-Whitney U test for non-normally distributed continuous variables. For continuous variables that showed statistically significant differences in the univariate analysis, we used the receiver operating characteristic (ROC) curve, calculated the area under the curve (AUC), and obtained the cutoff value. From the calculated cutoff values, the continuous variables were converted into binary variables. Next, univariate logistic regression analysis was conducted on the explanatory variables for which significant differences were obtained, and the variables were selected according to their statistical significance levels. Finally, multivariate logistic regression analysis was conducted on the explanatory variables that were significantly different in the univariate logistic regression analysis, and statistical significance was examined. Statistical analyses were performed using EZRver1.66 (Jichi Medical University, Saitama, Japan) and IBM SPSS Statistics for Windows, Version 29 (Released 2022; IBM Corp., Armonk, New York, United States), and the statistical significance was set at *p*<0.05 [12].

## Results

Patient selection process

During the study period, 156 patients underwent spinal surgery under general anesthesia. The exclusion criteria were as follows: zero patients were under 20 years of age, two did not undergo spinal surgery under general anesthesia, 44 were admitted to the ICU postoperatively, zero had an ASA-PS score of 4 or higher, three underwent emergency surgery, and seven underwent multilevel spine fusion surgery. A total of 56 patients were excluded from this study. Ultimately, 100 patients were included in the analysis. Of these, 50 patients were classified into the OUT and INT groups for analysis (Figure 1).

Preoperative factors

Preoperative factors are shown in Table 1. The OUT group had a higher proportion of diseases and procedures in the cervical spine, whereas the INT group had a higher proportion of diseases and procedures in the lumbar spine (p=0.013). Blood test data immediately before surgery showed a slight increase in PT in the INT group (p<0.001).

**Table 1:** Preoperative factors. BMI: Body mass index; TP: total protein; Alb: albumin; Hb: hemoglobin; Hct: hematocrit; Cre: creatinine; eGFR: estimated glomerular filtration rate; AST: aspartate aminotransferase; ALT: alanine aminotransferase; WBC: white blood cell; Plt: platelet; PT: prothrombin time; APTT: activated partial thromboplastin time; FDP: fibrinogen degradation products; CRP: C-reactive protein, *: *p*<0.05

		OUT group (n=50)	INT group (n=50)	Statistical analysis	Type of values	Statistical values	p-value
Site of lesion (%)	Cervical vertebrae	7(14)	16(32)	Fisher's exact test	n(%)	-	0.013*
	Thoracic vertebrae	4(8)	0(0)	-	n(%)	-	-
	Lumbar vertebrae	39(78)	34(68)	-	n(%)	-	-
Age(year)	-	69(22-84)	68.5(21-84)	Mann-Whitney U test	median (min-max)	1342.0	0.439
Sex	Men	32(64)	33(66)	Fisher's exact test	n(%)	-	＞0.999
	Women	18(36)	17(34)	-	n(%)	-	-
Height(cm)	-	164.0(137-203)	164.1(145.3-192)	Mann-Whitney U test	median(min-max)	1152.0	0.501
Body weight(kg)	-	64.1(38-120)	66.3(145.3-192)	Mann-Whitney U test	median(min-max)	1091.5	0.692
BMI(kg/m²)	-	24.42(16.23-37.07)	24.01(16.45-31)	Mann-Whitney U test	median(min-max)	1199.0	0.987
TP(g/dl)	-	7.0(6.1-8.0)	6.9(5.1-8.8)	Mann-Whitney U test	median(min-max)	1218.5	0.888
Alb(g/dl)	-	4.3(3.7-5.8)	4.3(2.5-5.3)	Mann-Whitney U test	median(min-max)	1372.5	0.440
Hb(g/dl)	-	13.8(10.6-17)	13.9(8.6-17.1)	Mann-Whitney U test	median(min-max)	1266.0	0.912
Hct(%)	-	40.9(32.3-48.7)	40.8(26.5-50.4)	Mann-Whitney U test	median(min-max)	1327.5	0.593
Cre(mg/dl)	-	0.80(0.4-1.8)	0.89(0.51-2.66)	Mann-Whitney U test	median(min-max)	1081.0	0.244
eGFR(ml/min/1.73m²)	-	66.8(28.7-120.6)	61.4(18.8-107.2)	Mann-Whitney U test	median(min-max)	1335.5	0.556
AST(U/L)	-	23(12-221)	23(12-41)	Mann-Whitney U test	median(min-max)	1272.5	0.320
ALT(U/L)	-	18(7-122)	21(6-53)	Mann-Whitney U test	median(min-max)	1160.5	0.537
WBC(/μl)	-	5.76(3.5-12.28)	6.25(4.1-9.7)	Mann-Whitney U test	median(min-max)	976.0	0.105
Plt(/μl)	-	218(110-379)	221(107-338)	Mann-Whitney U test	median(min-max)	1112.5	0.343
PT(sec)	-	11.5(10.5-13.8)	12.2(11-24.2)	Mann-Whitney U test	median(min-max)	623.5	<0.001*
APTT(sec)	-	29.1(24.1-51.9)	29.5(23.1-46.4)	Mann-Whitney U test	median(min-max)	1076.0	0.280
FDP(mg/dl)	-	291.6(201.6-612.5)	304(215.1-539.5)	Mann-Whitney U test	median(min-max)	1166.5	0.446
CRP(mg/dl)	-	0.05(0.01-4.27)	0.1(0.04-7.67)	Mann-Whitney U test	median(min-max)	556.0	0.057

Intraoperative factors

Intraoperative factors are shown in Table 2. There was no significant difference in ASA-PS between the two groups (p=0.498). In terms of the anesthesia method, inhalation anesthesia was more frequently used in the OUT group, while TIVA was more frequently used in the INT group (p<0.001). The mean operative time in the OUT group was 2.27 h (0.60-4.67h) and in the INT group, it was 1.67 h (0.57-5.83h) (p=0.001). Intraoperative drug use was significantly higher in the INT group for fentanyl (p<0.001), propofol (p<0.001), and tramadol (p=0.012). For intraoperative pain management, a higher percentage of patients in the OUT group received multiple-drug combinations. In contrast, a higher proportion of patients in the INT group received acetaminophen (p<0.001).

**Table 2:** Intraoperative factors. ASA-PS: American Society of Anesthesiologists Physical Status; TIVA: total intravenous anesthesia; NSAIDs: non-steroidal anti-inflammatory drugs; IV-PCA: intravenous patient-controlled analgesia, *: *p*<0.05

		OUT group (n=50)	INT group (n=50)	Statistical analysis	Type of values	Statistical values	p-value
ASA-PS(%)	1	8(16)	4(8)	Fisher's exact test	n(%)	-	0.498
	2	38(76)	40(80)	-	n(%)	-	-
	3	4(8)	6(12)	-	n(%)	-	-
Anesthesia method(%)	TIVA	2(4)	50(100)	Fisher's exact test	n(%)	-	<0.001*
	Inhalation anesthesia	46(86)	0(0)	-	n(%)	-	-
	Other	2(4)	0(0)	-	n(%)	-	-
Operation time(hours)	-	2.27(0.6-4.67)	1.64(0.57-5.83)	Mann-Whitney U test	median(min-max)	1738.0	0.001*
In/Out balance(ml)	-	880(250-995)	802.5(270-1795)	Mann-Whitney U test	median(min-max)	1441.5	0.187
Propofol(mg)	-	208.10 (282.88)	992.20 (363.10)	t-test	average(SD)	12.0	<0.001*
Remifentanil(mg)	-	2.54 (1.50)	2.65 (1.49)	t-test	average(SD)	0.3	0.714
Fentanyl(µg)	-	265.60 (118.08)	370.00 (136.18)	t-test	average(SD)	4.0	<0.001*
Rocuronium(mg)	-	73.18 (26.98)	41.90 (9.84)	t-test	average(SD)	-7.7	<0.001*
Tramadol(%)	Yes	7(14)	0(0)	Fisher's exact test	n(%)	-	0.012*
	No	43(86)	50(100)	-	n(%)	-	-
Intraoperative analgesia(%)	No drugs	2(4)	16(32)	Fisher's exact test	n(%)	-	<0.001*
	Acetaminophen single-drug	16(32)	28(56)	-	n(%)	-	-
	NSAID single-drug	1(2)	3(6)	-	n(%)	-	-
	Morphine single-drug	0(0)	0(0)	-	n(%)	-	-
	Multidrug combination	31(62)	3(6)	-	n(%)	-	-
IV-PCA(%)	Yes	47(94)	40(80)	Fisher's exact test	n(%)	-	0.071
	No	3(6)	10(20)	-	n(%)	-	-

Immediate postoperative factors (within 30 min after surgery)

The immediate postoperative factors, evaluated within 30 min of surgery, are shown in Table 3. A significantly higher percentage of patients in the OUT group reported pain within 30 min of surgery (p<0.001). For immediate postoperative pain management, a higher percentage of patients in the OUT group were administered NSAIDs. In contrast, a higher percentage of patients in the INT group received acetaminophen, morphine, or multidrug combinations (p<0.001).

**Table 3:** Postoperative factors. SpO2: Saturation of percutaneous oxygen; NRS: Numerical Rating Scale (modified); NSAIDs: non-steroidal anti-inflammatory drugs, *: *p*<0.05

		OUT group (n=50)	INT group (n=50)	Statistical analysis	Type of values	Statistical values	p-value
Immediate postoperative factors (within 30 minutes after surgery)							
Pain after surgery(%)	Yes	41(82)	15(30)	Fisher's exact test	n(%)	-	<0.001*
	No	9(18)	35(70)	-	n(%)	-	-
Pain management(%)	No drugs	34(68)	43(86)	Fisher's exact test	n(%)	-	0.001*
	Acetaminophen single-drug	0(0)	2(4)	-	n(%)	-	-
	NSAID single-drug	11(22)	1(2)	-	n(%)	-	-
	Morphine single-drug	0(0)	2(4)	-	n(%)	-	-
	Multidrug combination	5(10)	2(4)	-	n(%)	-	-
Adverse events(%)	Yes	24(48)	17(34)	Fisher's exact test	n(%)	-	0.222
	No	26(52)	33(66)	-	n(%)	-	-
Medical intervention(%)	Yes	20(40)	12(24)	Fisher's exact test	n(%)	-	0.133
	No	30(60)	38(76)	-	n(%)	-	-
Postoperative factors							
Length of hospital stay(day)	-	22(9-59)	16.5(7-64)	Mann-Whitney U test	median(min-max)	1722.0	0.032*
Body temperature(℃)	-	36.45(0.54)	36.48(0.35)	t-test	average(SD)	0.3	0.742
Pulse rate(count)	-	75.92(13.48)	72.82(12.20)	t-test	average(SD)	-1.2	0.231
Mean blood pressure(mmHg)	-	93.33(68-118)	100.33(68.67-138）	Mann-Whitney U test	median(min-max)	994.5	0.078
Respiratory rate(count)	-	12.22(2.64)	12.66(3.07)	t-test	average(SD)	0.7	0.444
SpO_2_(%)	-	98.52(1.27)	98.48(1.11)	t-test	average(SD)	-0.1	0.867
NRS(%)	0	4(8)	4(8)	Fisher's exact test	n(%)	-	0.288
	1	5(10)	1(2)	-	n(%)	-	-
	2	12(24)	7(14)	-	n(%)	-	-
	3	10(20)	18(36)	-	n(%)	-	-
	4	13(26)	14(28)	-	n(%)	-	-
	5	6(12)	6(12)	-	n(%)	-	-
Postoperative pain(%)	Yes	46(92)	47(94)	Fisher's exact test	n(%)	-	>0.999
(within 24 hours after surgery)	No	4(8)	3(6)	-	n(%)	-	-
Postoperative pain management(%)	No drugs	11(22)	22(44)	Fisher's exact test	n(%)	-	<0.001*
(within 24 hours after surgery)	Acetaminophen single-drug	0(0)	12(24)	-	n(%)	-	-
	NSAID single-drug	11(22)	5(10)	-	n(%)	-	-
	Morphine single-drug	0(0)	0(0)	-	n(%)	-	-
	Multidrug combination	28(56)	11(22)	-	n(%)	-	-
Analgesics used up to 24 hours after surgery							
Acetaminophen(mg)	-	1000(0-2400)	1000(0-3000)	Mann-Whitney U test	median(min-max)	1053.0	0.090
NSAIDs(mg)	-	72.00 (41.54)	19.5(31.66)	t-test	average(SD)	0.03	<0.001*
Tramadol(mg)	-	14(40.46)	2(14.14)	t-test	average(SD)	0.01	0.051
Pentazocine(mg)	-	15(0-45)	0(0-30)	Mann-Whitney U test	median(min-max)	1738.0	<0.001*

Postoperative factors

Postoperative factors are shown in Table 3. The mean length of hospital stay was 22 days (9-59 days) in the OUT group and 16.5 days (7-64 days) in the INT group (p=0.032). There was no statistically significant difference in vital signs between the two groups immediately after returning to the ward. Almost all patients in both groups experienced postoperative pain, and the maximum NRS score remained high at 3-4 points. For postoperative pain management, a higher percentage of patients in the OUT group received multi drugs combination. In contrast, the INT group had a higher proportion of patients who did not receive any drugs (p<0.001).

Analgesics used up to 24 h after surgery

The analgesics used up to 24 h after surgery are shown in Table 3. The OUT group used more NSAIDs and pentazocine (p<0.001) compared with those used by the other group.

Independent factors associated with the introduction of MEP monitoring

**Figure 2:**
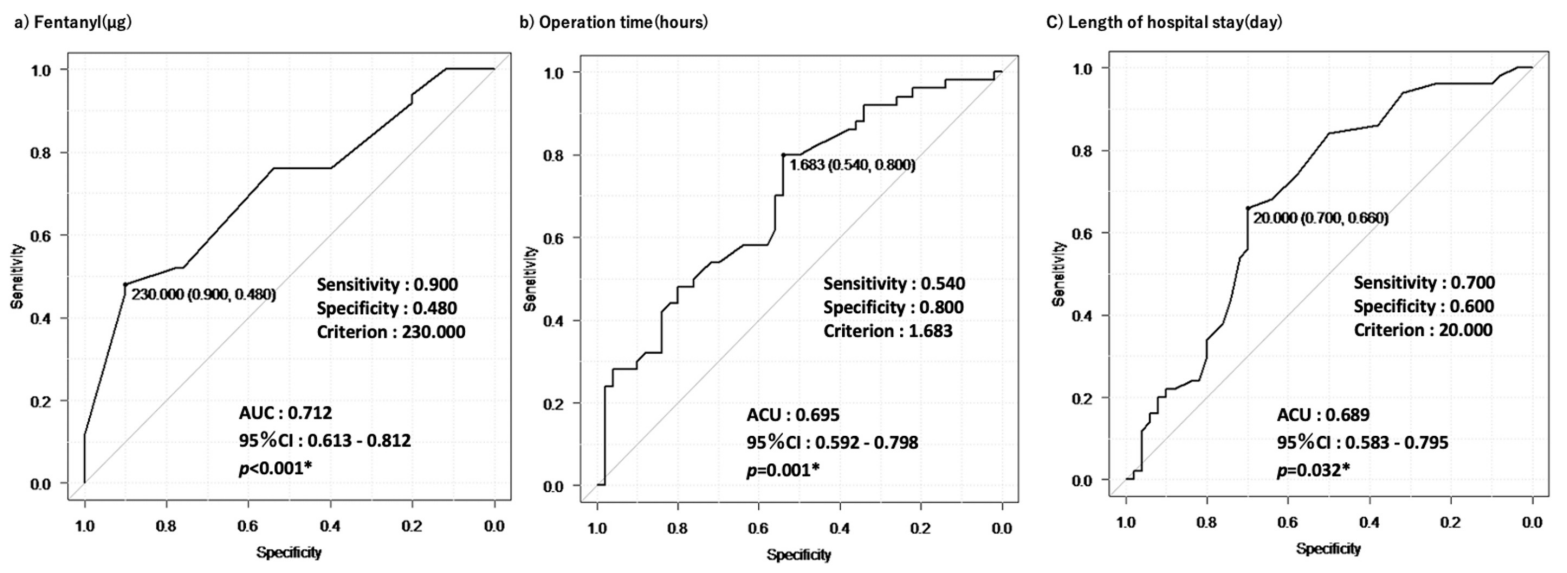
ROC curves generated by a) Fentanyl (μg), b) Operation time (hours), and c) Length of hospital stay (days). *: p<0.05

ROC curves were used for continuous variables that were significantly different in univariate analysis. Cutoff values were calculated for three items: intraoperative fentanyl dose, operation time, and length of hospital stay, and the high- and low-value groups were classified as binary variables. The cutoff values, sensitivity, specificity, AUC, and 95% confidence interval (CI) for each item are shown in Figure 2. A total of 13 items showed significant differences between the two groups in univariate analysis and were entered as explanatory variables. Univariate logistic regression analysis revealed statistically significant differences in six items: intraoperative fentanyl dose, intraoperative analgesia, operation time, length of hospital stay, postoperative pain within 30 min, and postoperative pain management. Multivariate logistic regression analysis showed that intraoperative fentanyl dose, intraoperative analgesia, operation time, postoperative pain within 30 min, hospital stay, and postoperative pain management were independent factors associated with the introduction of MEP monitoring (Table 4). Compared with the OUT group, the INT group showed a significantly lower intraoperative fentanyl dosage (OR: 0.111, 95% CI: 0.0177-0,696, p=0.0189), a significantly lower proportion of intraoperative non-narcotic analgesic measures (OR: 4.46, 95% CI: 2.01-9.88, p<0.001), and a significantly shorter operative time (OR: 6.1, 95% CI: 1.06-35.2, p=0.0432). The INT group significantly had fewer patients complaining of postoperative pain within 30 min (OR: 12.5, 95% CI: 2.55-61.2, p=0.0186) and requiring analgesic measures within 24 h after surgery (OR: 1.85, 95% CI: 1.05-3.26, p=0.0334). Length of hospital stay was also significantly reduced in the INT group (OR: 5.49, 95% CI: 1.2-25.1, p=0.0282).

**Table 4:** Logistic regression analysis with MEP monitoring introduction as the objective variable. *: *p*<0.05, **: Percentage of people for whom pain management was performed MEP: Motor-evoked potential

	Univariate analysis				Multivariable logistic regression		
	Odds ratio	95％CI	p-value		Odds ratio	95％CI	p-value
Fentanyl(µg)	0.12	0.041-0.354	<0.001*		0.111	0.0177-0.696	0.0189*
Intraoperative analgesia(%)**	3.92	2.33-6.61	<0.001*		4.46	2.01-9.88	0.00023*
Operation time（hours）	4.16	1.74-9.94	<0.01*		6.1	1.06-35.2	0.0432*
Pain management (%)**	10.6	4.15-27.3	<0.001*		12.5	2.55-61.2	0.00186*
(within 30 minutes after surgery)							
Length of hospital stay(day)	2.97	1.3-6.76	<0.01*		5.49	1.2-25.1	0.0282*
Postoperative pain management (%)**	1.92	1.37-2.7	<0.001*		1.85	1.05-3.26	0.0334*
(within 24 hours after surgery)							

## Discussion

In spinal surgery, MEP monitoring and anesthesia management with TIVA are recommended for the prevention and early detection of nerve damage. This study aimed to determine whether MEP monitoring contributes to a reduction in the length of hospital stay and postoperative acute pain in patients undergoing spinal surgery under general anesthesia.

Introduction of MEP monitoring and surgical time

Previous studies have not mentioned whether the introduction of MEP monitoring has a direct impact on operative time [1-7]. In the present study, the mean operative times in the OUT group were 2.27 h and 1.64 h in the INT group. The operative time was significantly shorter in the INT group, indicating that the introduction of MEP monitoring had an impact on operation time (OR: 6.1, 95% CI: 1.06-35.2, *p*=0.0432). In spinal surgery, one of the most critical complications to avoid by a surgeon is motor disability due to postoperative nerve palsy [13-15]. Generally, MEP monitoring is performed to improve the efficiency and safety of spine surgery [16], and the introduction of MEP monitoring may reduce the operating time by streamlining the surgeon's technique during spine surgery and enabling safe nerve processing. MEP monitoring is believed to shorten operation time, reduce anesthesia and wound opening times, and allow for less invasive surgical procedures.

Increase in intraoperative fentanyl consumption due to changes in the anesthesia method

Previous studies comparing volatile inhalation anesthetics and intravenous anesthetics did not report any changes in intraoperative fentanyl consumption [17-19]. However, in the present study, intraoperative fentanyl consumption increased significantly in the INT group that underwent TIVA (265.6μg in the OUT group vs. 370μg in the INT group, OR: 0.111, 95% CI: 0.0177-0,696, *p*=0.0189). The intravenous anesthetics used in TIVA, such as propofol, do not have analgesic or muscle-relaxant effects. Therefore, during TIVA, a combination of analgesics, adjunctive analgesics, and muscle relaxants is recommended based on the patient’s needs [20-22]. In spine surgery, unintended arousal or body movements during the procedure can lead to serious complications, including nerve damage [23]. Spinal surgery requires close attention to intraoperative arousal and immobilization during anesthesia. However, muscle relaxants cannot be administered intraoperatively during MEP monitoring, as they suppress MEP signals [7-11]. Therefore, in TIVA, additional analgesics should be administered as needed to prevent unintended intraoperative arousal or body movements due to the increased pain associated with the operation. Narcotic analgesics such as remifentanil and fentanyl were the primary choices in this case [24]. Remifentanil offers excellent analgesic properties and precise drug modulation. In contrast, high doses of remifentanil exacerbate postoperative pain due to hyperalgesia. In TIVA, intravenous fentanyl, which has comparable potency to remifentanil, may serve as an effective analgesic [25]. In this study, the intraoperative dose of fentanyl was increased in the INT group. These findings suggest that in TIVA, intraoperative opioid administration may be adjusted to minimize unintended intraoperative arousal and body movements, with anesthesia management prioritizing analgesia.

Reduction in postoperative pain and length of hospital stay

In this study, a significant reduction in hospital stay was observed in the INT group (22 days in the OUT group vs. 16.5 days in the INT group, OR: 5.49, 95% CI: 1.2-25.1, *p*=0.0282). In previous studies, postoperative pain has been implicated in patient outcomes such as the length of hospital stay. Appropriate postoperative pain relief is believed to promote early release from hospital and rehabilitation programs, resulting in shorter hospital stays [26-28]. In this study, the proportion of patients complaining of postoperative pain within 30 min after surgery was significantly lower in the INT group (OUT group: 41 vs. INT group: 9, OR: 12.5, 95% CI: 2.55-61.2, *p*=0.0186), and the proportion of patients who took analgesic measures within 24 h after surgery was also lower (OUT group: 39 vs. INT group: 28, OR: 1.85, 95% CI: 1.05-3.26, *p*=0.0334). In addition, the total dose of analgesics required postoperatively within 24 h in the INT group was clearly reduced (Table 4). These results suggested that acute pain occurred less frequently in the INT group between 30 min and 24 h postoperatively. The decreased frequency of acute postoperative pain may have contributed to the early weaning of patients and rehabilitation programs, resulting in a shorter length of hospital stay. The intraoperative dose of fentanyl was significantly increased in the INT group (265.6μg in the OUT group vs. 370 μg in the INT group, OR: 0.111, 95% CI: 0.0177-0,696, *p*=0.0189). In the INT group, a change in the anesthetic method to TIVA increased the intraoperative opioid dose and reduced the acute postoperative pain [29]. These results suggest that the introduction of MEP monitoring and changes in anesthesia methods may have influenced the reduction in postoperative acute pain and effectively shortened the length of hospital stay.

Limitations

This study has several limitations that require careful interpretation. First, this was a single-center retrospective cross-sectional study. The results and interpretations are subject to numerous biases because of the small population size and the retrospective analysis of a limited number of measures. Second, this study was based on a small sample of subjects and time periods; there is a six-year gap between the two groups, and the results were expected to differ significantly depending on the primary disease, surgical technique, and patient background. The data for each endpoint were collected up to 24 h postoperatively, and it was necessary to evaluate the events that occurred 24 h postoperatively in detail. Events that occur during a patient’s hospital stay require detailed data collection and evaluation. Third, this study was conducted in patients undergoing spine surgery, and MEP monitoring and TIVA are techniques that have been widely applied in non-spinal surgery patients. Given the specificity of the surgical population, it is unlikely that these results can be extrapolated to patients undergoing non-spinal procedures. Therefore, careful interpretation is warranted. The results of this study are unlikely to be similar in patients who have undergone non-spinal surgery and should be carefully considered.

## Conclusions

This study aimed to determine whether the introduction of MEP monitoring contributes to a reduction in the length of hospital stay and acute postoperative pain in patients undergoing spinal surgery under general anesthesia. MEP monitoring may improve the efficiency of the surgeon's technique in spinal surgery and shorten the operative time. In addition, the decreased frequency of acute pain in the INT group may have promoted early postoperative release from the hospital and rehabilitation programs, and as a result, contributed to a shorter hospital stay. Acute postoperative pain in the INT group may have been influenced by the intraoperative fentanyl dose.
